# Application of Multiplexed Kinase Inhibitor Beads to Study Kinome Adaptations in Drug-Resistant Leukemia

**DOI:** 10.1371/journal.pone.0066755

**Published:** 2013-06-24

**Authors:** Matthew J. Cooper, Nathan J. Cox, Eric I. Zimmerman, Brian J. Dewar, James S. Duncan, Martin C. Whittle, Thien A. Nguyen, Lauren S. Jones, Sreerupa Ghose Roy, David M. Smalley, Pei Fen Kuan, Kristy L. Richards, Richard I. Christopherson, Jian Jin, Stephen V. Frye, Gary L. Johnson, Albert S. Baldwin, Lee M. Graves

**Affiliations:** 1 Department of Pharmacology, School of Medicine, University of North Carolina, Chapel Hill, North Carolina, United States of America; 2 Lineberger Comprehensive Cancer Center, University of North Carolina, Chapel Hill, North Carolina, United States of America; 3 Curriculum in Genetics & Molecular Biology, University of North Carolina, Chapel Hill, North Carolina, United States of America; 4 Department of Biology, University of North Carolina, Chapel Hill, North Carolina, United States of America; 5 Department of Biostatistics, Gillings School of Global Public Health, University of North Carolina, Chapel Hill, North Carolina, United States of America; 6 Division of Hematology & Oncology, Department of Medicine, School of Medicine, University of North Carolina, Chapel Hill, North Carolina, United States of America; 7 School of Molecular Bioscience, University of Sydney, Sydney, New South Wales, Australia; 8 Center for Integrative Chemical Biology and Drug Discovery, Division of Chemical Biology and Medicinal Chemistry, Eshelman School of Pharmacy, University of North Carolina, Chapel Hill, North Carolina, United States of America; University of Virginia, United States of America

## Abstract

Protein kinases play key roles in oncogenic signaling and are a major focus in the development of targeted cancer therapies. Imatinib, a BCR-Abl tyrosine kinase inhibitor, is a successful front-line treatment for chronic myelogenous leukemia (CML). However, resistance to imatinib may be acquired by BCR-Abl mutations or hyperactivation of Src family kinases such as Lyn. We have used multiplexed kinase inhibitor beads (MIBs) and quantitative mass spectrometry (MS) to compare kinase expression and activity in an imatinib-resistant (MYL-R) and -sensitive (MYL) cell model of CML. Using MIB/MS, expression and activity changes of over 150 kinases were quantitatively measured from various protein kinase families. Statistical analysis of experimental replicates assigned significance to 35 of these kinases, referred to as the MYL-R kinome profile. MIB/MS and immunoblotting confirmed the over-expression and activation of Lyn in MYL-R cells and identified additional kinases with increased (MEK, ERK, IKKα, PKCβ, NEK9) or decreased (Abl, Kit, JNK, ATM, Yes) abundance or activity. Inhibiting Lyn with dasatinib or by shRNA-mediated knockdown reduced the phosphorylation of MEK and IKKα. Because MYL-R cells showed elevated NF-κB signaling relative to MYL cells, as demonstrated by increased IκBα and IL-6 mRNA expression, we tested the effects of an IKK inhibitor (BAY 65-1942). MIB/MS and immunoblotting revealed that BAY 65-1942 increased MEK/ERK signaling and that this increase was prevented by co-treatment with a MEK inhibitor (AZD6244). Furthermore, the combined inhibition of MEK and IKKα resulted in reduced IL-6 mRNA expression, synergistic loss of cell viability and increased apoptosis. Thus, MIB/MS analysis identified MEK and IKKα as important downstream targets of Lyn, suggesting that co-targeting these kinases may provide a unique strategy to inhibit Lyn-dependent imatinib-resistant CML. These results demonstrate the utility of MIB/MS as a tool to identify dysregulated kinases and to interrogate kinome dynamics as cells respond to targeted kinase inhibition.

## Introduction

The constitutively active BCR-Abl tyrosine kinase is the product of the reciprocal translocation of chromosomes 9 and 22 and the causative oncoprotein in over 95% of chronic myeloid leukemia (CML) cases [1]. Imatinib (Gleevec™), a small molecule ATP-competitive inhibitor of BCR-Abl, is an effective front-line treatment for CML and has established the concept of targeted kinase inhibition as a viable strategy for cancer therapy [2]. However, whereas the majority of newly diagnosed CML patients undergo remission, some patients are refractory to imatinib therapy and others who initially respond will eventually develop imatinib resistance [3]–[5].

Multiple mechanisms of cellular resistance to imatinib have been described and include BCR-Abl-dependent mechanisms such as protein overexpression or expression of inhibitor-resistant mutations in the BCR-Abl kinase domain, such as the T315I “gatekeeper mutation” [6]. This mutation reduces the affinity of tyrosine kinase inhibitors while increasing the leukemogenic signaling of BCR-Abl [7]–[9]. Resistance also arises from BCR-Abl-independent mechanisms such as alterations in drug import or export that affect intracellular imatinib levels [10]–[13], clonal evolution as the result of additional genetic abnormalities [14], [15], and upregulation of alternative signaling pathways [8], [16].

Upregulation of kinases such as Akt or Src family kinases (SFKs) have been implicated in imatinib resistance whereby these kinases drive alternative cell survival and proliferation signaling [6], [17]–[20]. For instance, hyper-activation of Lyn or Hck has been associated with imatinib resistance in CML patients and cell culture models [21]–[24], albeit the mechanisms by which these kinases contribute to imatinib resistance is not well understood. Moreover, a recent study reported that SFKs are frequently involved in promoting inhibitor-resistant CML, even after successful inhibition of BCR-Abl activity [25].

Large-scale proteomics studies have analyzed differential protein expression and phosphorylation in drug-resistant leukemia [26]–[29]. The expression and activation state of protein kinases (i.e., the kinome) may contribute significantly to the cellular adaptation to drug resistance, and recent technologies have been developed to study the kinome *en masse*. One such technology is the development of kinase inhibitor-conjugated beads used for the enrichment of protein kinases [30], [31]. These broad spectrum kinase inhibitors target the ATP-binding pocket and allow the unbiased capture of kinases, including low abundant kinases. Quantitative methods of mass spectrometry (e.g., iTRAQ and SILAC) are subsequently applied to evaluate kinome changes on a global scale [30]–[34]. We have previously applied a methodology using multiplexed kinase inhibitor-conjugated beads (MIBs) combined with methods of quantitative mass spectrometry (MIB/MS) to examine kinome reprogramming in triple-negative breast cancer in response to MEK inhibition [35].

In this study, we applied the MIB/MS strategy to investigate kinome adaptations in a cell line model of Lyn-driven, imatinib-resistant CML (MYL-R) and compared this to its imatinib-sensitive counterpart (MYL) [22]. Quantitative proteomic profiling by iTRAQ and LC TEMPO MALDI-TOF/TOF identified multiple kinome differences in the imatinib-resistant MYL-R cells. These differences included upregulation of Lyn, as well as kinases involved in the MEK/ERK (MEK2, ERK2) and NF-κB (IKKα) pathways. We found that pharmacological and RNAi-mediated inhibition of Lyn reduced the activity of MEK2 and IKKα, implicating these pathways as mediators of Lyn-driven imatinib resistance.

Furthermore, we used the MIB/MS strategy to examine the kinome responses of MYL-R cells to targeted inhibition of MEK and IKK. The kinome response profiles indicated that targeted inhibition of these kinases led to upregulation of pro-survival kinases, however combined inhibition prevented this response. Moreover, the combined targeted inhibition of MEK and IKK successfully overcame MYL-R drug resistance and led to significantly reduced cell viability and induction of apoptosis. Thus, this study demonstrates that MIB/MS kinome profiling is a powerful tool both for detecting dysregulated kinases and for identifying targeted therapies for the treatment of drug-resistant leukemia.

## Results

### Use of Multiplexed Inhibitor Beads (MIBs) to Analyze the Kinomes of Drug-sensitive and -resistant Leukemia Cells

We applied the MIB/MS methodology described previously [35] to examine differences in the kinomes between an imatinib-resistant, Lyn-dependent CML cell line (MYL-R) and its imatinib-sensitive counterpart (MYL) [22]. Kinases were isolated from MYL and MYL-R cell lysates by MIBs enrichment, which captures a broad range of kinases as a function of their expression level and activation state [31], [35]–[37]. Kinase expression and activity was quantitatively profiled by labeling trypsin-digested MIB eluates with iTRAQ isobaric mass tags, and analyzing labeled peptides by MALDI TOF/TOF mass spectrometry (MIB/MS) as described earlier [35].

**Figure 1:**
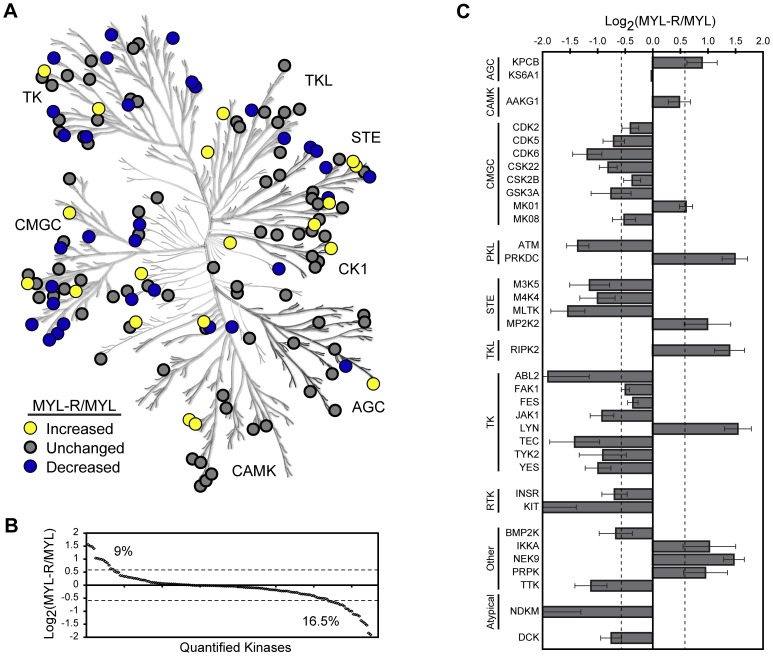
Application of MIB/MS to analyze the kinomes of drug-sensitive and -resistant leukemia cells. Kinases from MYL and MYL-R cells were affinity enriched with multiplexed inhibitor beads and quantified by LC-MALDI TOF/TOF mass spectrometry (MIB/MS). Results from three independent experiments were pooled as described in Materials and Methods. (**A**) Over 150 kinases were quantified from MYL-R relative to MYL cells across a broad spectrum of kinase families, as depicted in the phylogenetic tree of the human protein kinase superfamily. Yellow and blue dots signify kinases that were increased or decreased in abundance, respectively, in MYL-R relative to MYL cells while gray dots signify kinases that were unchanged. Kinome illustration reproduced courtesy of Cell Signaling Technology, Inc. (www.cellsignal.com). (**B**) The trend of kinase abundance changes for all kinases quantified. *Dashed line*, ±1.5-fold change. (**C**) The kinome profile of MYL-R relative to MYL was derived from the kinases that were significantly changed after statistical analysis. The kinase abundance ratios and p-values from three independent experiments were combined and adjusted for multiple hypothesis testing and ratios with a Benjamini-Hochberg q-value <0.2 were considered significant. *Dashed lines*, ±1.5-fold change; *error bars*, SE (N = 3).

The MS/MS spectra from three independent MIB/MS experiments were analyzed by ProteinPilot™ (AB Sciex, Framingham, MA) using the ProGroup™ algorithm for protein identification. We identified a total of 165 kinases with >95% confidence, (ProteinPilot™ Unused ProtScore >1.3), representing each protein kinase group and several non-protein kinases involved in regulating metabolic processes (Figure 1A, Table S1). Over 150 of these kinases were quantified, revealing multiple changes in kinase abundance in MYL-R compared to MYL cells (Figure S1A, Table S1). To visualize the trend in kinase abundance changes we set a cutoff of ±1.5-fold; this threshold was based on previous analysis of technical replicates in our lab, using guidelines proposed by Unwin, et al. [38]. According to these criteria, ≈ 9% of quantified kinases were increased in abundance in MYL-R cells and nearly twice as many were decreased while the majority of kinases remained unchanged (Figure 1B).

As a complement to iTRAQ quantification, we performed a MIB/MS experiment comparing SILAC-labeled MYL and MYL-R cells. As shown in Figure S1B, S1C, both quantification methods revealed similar trends in kinase profiles, in terms of kinase identity and direction of change. Quantification by SILAC showed that ≈ 22% of kinases were increased in abundance in MYL-R cells while ≈ 16% of kinases were decreased. This observation agrees with previous reports that iTRAQ tends to underestimate differences in protein abundance [39], [40].

In order to establish the kinome profile for MYL-R relative to MYL cells, we pooled the kinase abundance ratios from three MIB/MS experiments, combined their p-values and then corrected for multiple hypothesis testing expressed as a q-value according to the Benjamini-Hochberg method [41]. Reasoning that MIB/MS is a discovery tool to be validated by other methods such as immunoblotting, we accepted all quantified kinases with a q-value <0.2. The relative abundances of the 35 kinases that met these criteria are referred to as the MYL-R kinome profile (Figure 1C). This profile shows that the kinases increased in MYL-R relative to MYL were: AMPK (AAKG1), DNA-PK (PRKDC), ERK2 (MK01), IKKα, Lyn, MEK2 (MP2K2), Nek9, PKCβ (KPCB), PRPK and RIPK2. Those decreased in MYL-R relative to MYL were: Abl2, ASK1 (M3K5), ATM, BMP2K, CDK2, CDK5, CDK6, CK2a’ (CSK22), CK2b (CSK2B), dCK, FAK1, Fes, GSK3a, HGK (M4K4), INSR, Jak1, JNK1 (MK08), c-Kit, MLTK, NDKM, RSK1 (KS6A1), Tec, TTK, Tyk2, Yes. These data demonstrate that the MIB/MS approach identified significant and quantitative differences between the kinomes of MYL and MYL-R cells.

### Validation of MIB/MS Kinome Results by Immunoblotting

**Figure 2:**
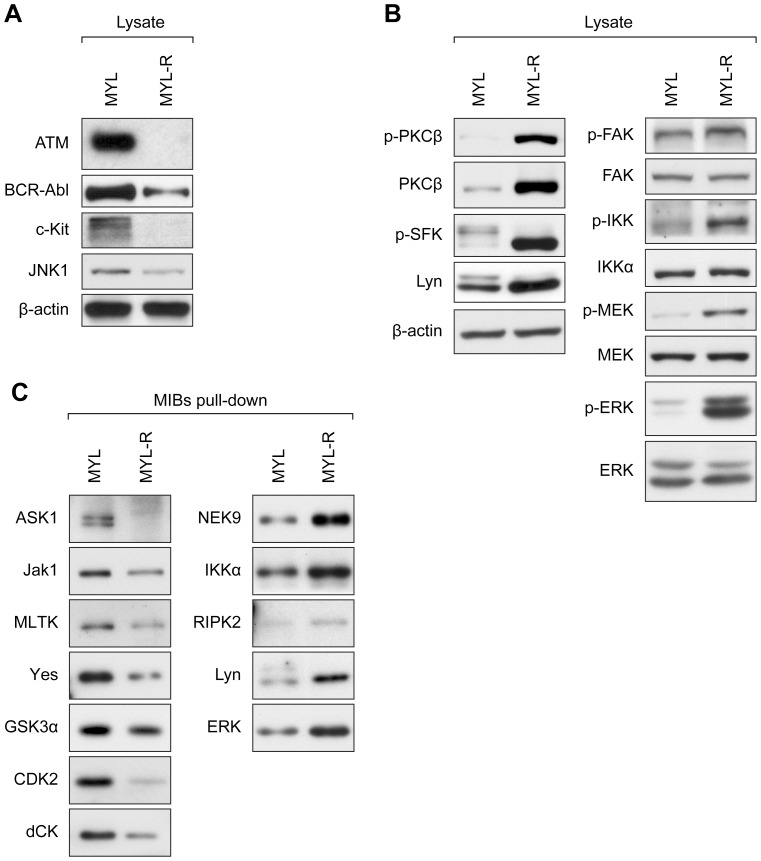
Validation of the MYL-R kinome profiles by immunoblotting. (**A and B**) MYL and MYL-R cell lysates were analyzed by immunoblot with the antibodies indicated. Representative data are shown. (**A**) ATM, BCR-Abl and c-Kit were decreased in MYL-R relative to MYL cells as predicted from the kinome profile above. (**B, left panel**) Both total and activated Lyn and PKCβ were increased in MYL-R cells as shown using antibodies to detect phosphorylation of the activation loop of Lyn (p-Y416) or the autophosphorylation site of PKCβ. (**B, right panel**) Total amounts of IKKα, MEK2 and ERK1/2 were similar in MYL and MYL-R cells, however, phospho-specific antibodies demonstrated these kinases were more active in MYL-R cells. (**C**) Kinases from MYL and MYL-R cell lysates were captured by MIBs pull-down and analyzed by immunoblot using antibodies directed against total protein. The relative amounts of kinases captured from each cell lysate correlated with the abundance ratios predicted by the MYL-R kinome profile. MIBs exposed to MYL-R cell lysate (**C, left panel**) captured less ASK1, Jak1, MLTK, Yes, GSK3α, CDK2 and dCK; and (**C, right panel**) captured more NEK9, IKKα, RIPK2, Lyn and ERK. See also, Figures S2 and S3.

To confirm changes in specific kinases detected by MIB/MS, lysates from MYL and MYL-R cells were analyzed by immunoblotting to examine the expression and phosphorylation of candidate kinases in the two cell lines. As shown in Figure 2A, reduced amounts of ATM, BCR-Abl, c-Kit and JNK1 protein were detected in MYL-R compared to MYL lysates, confirming the lower total expression of these kinases detected by MIB/MS analysis. By contrast, kinases detected as increased by MIB/MS (PKCβ, Lyn, FAK1, IKKα, MEK2, and ERK2) were observed to have greater expression or activation loop phosphorylation in MYL-R cells, reflecting the ability of MIBs to capture kinases based both on abundance and activity [35]. Immunoblot analysis showed that Lyn and PKCβ protein was higher in MYL-R compared to MYL (Figure 2B, left panel), whereas the total amount of FAK1, IKKα, MEK2 and ERK2 was comparable between the two cell lines (Figure 2B, right panel). Interestingly, immunoblot analysis using antibodies to detect the phosphorylation of the activation loops (IKKα, MEK2 and ERK2) or autophosphorylation sites (Lyn, PKCβ, FAK1) indicated increased activation of each of these kinases in the MYL-R samples.

We further investigated the activity-dependent binding of kinases to MIBs by performing pull-down assays to compare the amount of kinases bound to MIBs with or without phosphatase treatment. MYL and MYL-R cell lysate was incubated with or without calf intestinal alkaline phosphatase, incubated with MIBs and bound proteins were eluted with SDS sample buffer and analyzed by immunoblot. Analysis of cell lysates (Figure S2, top) showed that treatment with alkaline phosphatase eliminated phospho-IKKα while levels of total IKKα were unaffected. Analysis of MIBs eluate for levels of total IKKα, Lyn and MEK2 (Figure S2, bottom) showed that a greater amount of these kinases were captured from MYL-R lysate than from MYL lysate, correlating with the increased kinase abundance and phosphorylation in MYL-R detected by MIB/MS (Figure 1C) and immunoblot analysis (Figure 2B). Treatment of MYL-R lysates with alkaline phosphatase eliminated the binding of IKKα, Lyn and MEK2 to MIBs, demonstrating the influence of kinase activity on MIB binding.

As demonstrated above, a limitation of MIB/MS for global kinome analysis is that validation by immunoblotting may require the use of high quality phospho-specific antibodies to distinguish between total kinase levels and activated kinase that is preferentially captured by MIBs. Therefore, to allow further validation using available pan-antibodies we performed MIBs pull-down assays to compare the amount of kinases captured from MYL and MYL-R cell lysates. Cell lysates were incubated with MIBs, bound kinases were eluted with SDS sample buffer, and eluates were analyzed by immunoblot using antibodies directed against total protein. We examined 12 kinases captured from MYL and MYL-R cell lysates and in each case the relative kinase amount corroborated the MYL-R kinome profile. The kinases observed to be decreased in MYL-R eluates were ASK1, Jak1, MLTK, Yes, GSK3α, CDK2 and dCK (Figure 2C, left panel), and the kinases observed to be increased in MYL-R eluates were NEK9, RIPK2, IKKα, Lyn and ERK (Figure 2C, right panel). The latter three kinases were included to verify that this approach would show differences among cell lines in kinases with changes in total expression (Lyn) as well as changes only in activation state (IKKα, ERK). Because the antibody used to detect phospho-IKKα also recognizes phospho-IKKβ, we separated proteins from cell lysates on a low-percentage polyacrylamide gel and performed immunoblot analysis to compare the migration of phospho-IKK with total IKKα and IKKβ (Figure S3). This data indicated a significant increase in the phosphorylation of IKKα, but not IKKβ, in MYL-R cells and confirmed the selective increase in IKKα binding detected by MIB/MS.

In total, we confirmed the expression level and activity changes of 19 of the 35 kinases found in the MYL-R kinome profile. These results thus demonstrate the ability of MIB/MS to detect specific changes in kinase abundance and activity, and validate it as a tool to profile changes in the kinome.

### Lyn kinase Drives Activation of MEK and Potentiates Activation of IKKα in MYL-R Cells

Applying MIB/MS to study kinome adaptations in MYL-R identified a significant increase in Lyn, a kinase associated with some types of imatinib-resistant CML [21], [22]. Consequently, we asked whether we could use the kinome profile of MYL-R cells to identify other kinases regulated by Lyn. Kinases increased in MYL-R cells include those in the MEK/ERK pathway [MEK2 (MP2K2) and ERK2 (MK01)] and the NF-κB pathway (IKKα), both of which could potentially be activated by Lyn [42]–[45].

**Figure 3:**
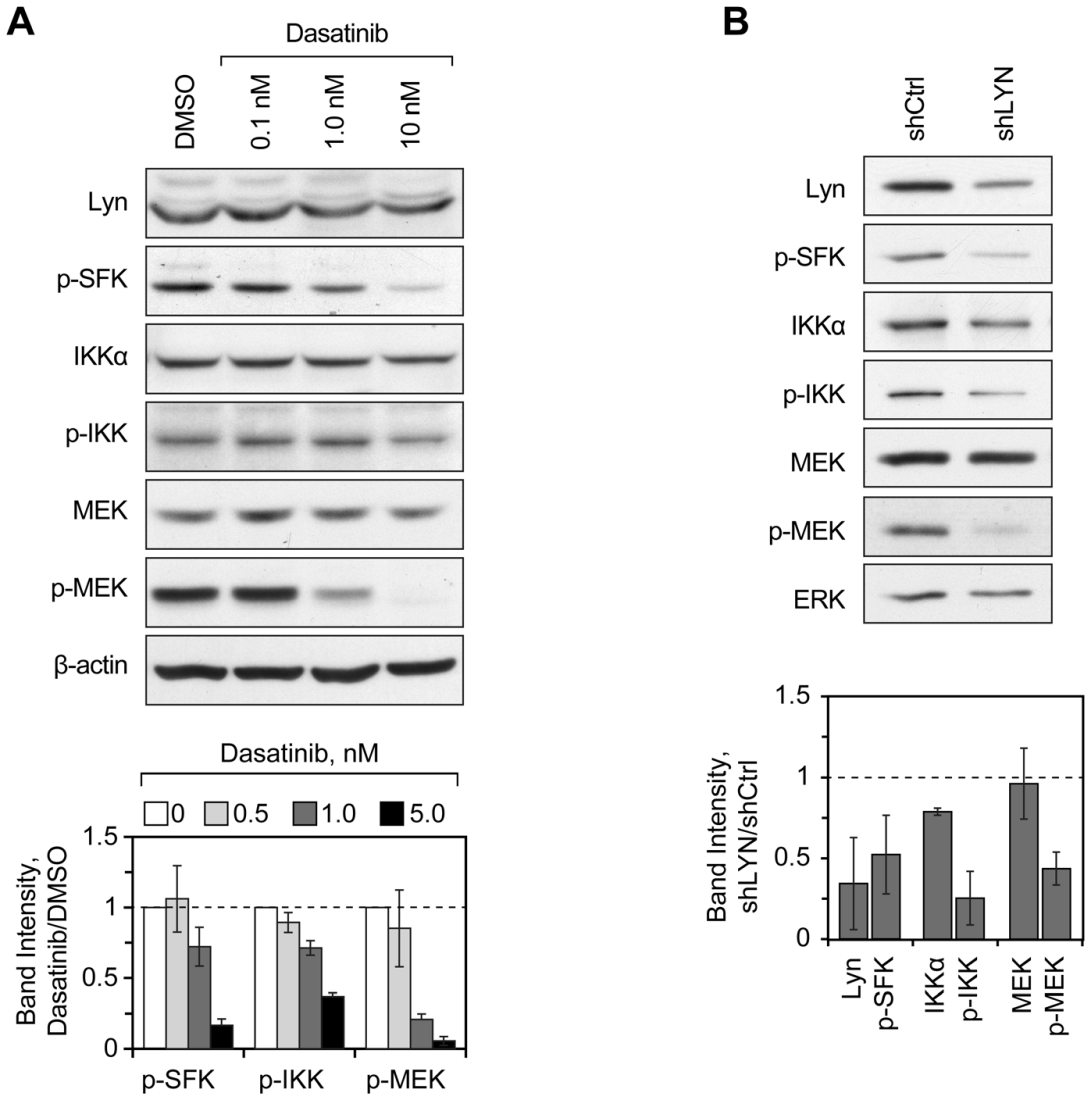
Lyn drives activation of MEK and increases activation of IKKα in MYL-R cells. (**A**) MYL-R cells were treated for 1 hour with dasatinib, as indicated, and lysates were analyzed by immunoblot with the antibodies indicated. Lyn phosphorylation was detected by p-SFK (Y416). For densitometry, the band densities of p-SFK, p-MEK and p-IKKα were normalized against the β-actin loading control and were plotted relative to the DMSO treatment. *Error bars*, SE (N = 3). (**B**) MYL-R cells were transduced with non-targeting shRNA (shCtrl) or shRNA directed against Lyn (shLyn) and lysates were analyzed by immunoblot with the antibodies indicated. Lyn phosphorylation was detected by p-SFK (Y416) antibody. For densitometry, the band densities of p-IKKα and p-MEK were plotted relative to total IKKα and MEK protein expression. *Error bars*, SE (N = 2).

In order to investigate the effect of Lyn on activation of these kinases, we treated MYL-R cells with varying amounts (0.1–10 nM) of the dual Abl/Src family kinase (SFK) inhibitor dasatinib for 1 hour and assessed the phosphorylation status of Lyn, MEK and IKKα by immunoblot analysis. As shown in Figure 3A, dasatinib treatment nearly abolished Lyn and MEK phosphorylation, while having a moderate effect on IKKα phosphorylation (≈ 60% reduction). These results suggest that Lyn is responsible for activation of MEK in MYL-R cells and that it may partially regulate IKKα activity.

We used MIB/MS to investigate the MYL-R kinome response to inhibition of Lyn by dasatinib. To test the initial kinome response to dasatinib, we treated MYL-R cells with dasatinib for 1 hour at 10 nM, a concentration sufficient to completely inhibit Lyn (data not shown), and then analyzed the lysates by MIB/MS as described earlier. Kinase abundance ratios were calculated for dasatinib treatment compared to DMSO control, resulting in the quantification of 139 kinases as shown in Figure S4 and Table S2. Kinases decreased after dasatinib treatment included established dasatinib targets such as the SFKs Lyn and Yes, Abl2, BTK and CSK [46]–[48]. Other kinases observed to be decreased have either not been reported as dasatinib targets, or have an affinity for dasatinib reported to be lower than 10 nM [32], [34], indicating that they may be indirect targets of dasatinib treatment. These included kinases in the MEK/ERK pathway [B-Raf, MEK2 (MP2K2), ERK2 (MK01), RSK1 (KS6A1)] as well as a kinase known to activate IKK (RIPK2) [49], [50].

Because of concerns about the selectivity of dasatinib, we performed Lyn knockdown using lentiviral shRNA as described in Material and Methods. As shown in Figure 3B, transduction of MYL-R cells with Lyn shRNA reduced total and phosphorylated Lyn by ≈ 65% and ≈ 50%, respectively. Lyn knockdown resulted in reduced phosphorylation of both MEK (≈ 57%) and IKKα (≈ 75%), as observed with dasatinib, supporting the involvement of Lyn signaling in the activation of MEK and IKKα in MYL-R cells.

### MYL-R Exhibit an Increased Level of NF-κB Signaling

**Figure 4:**
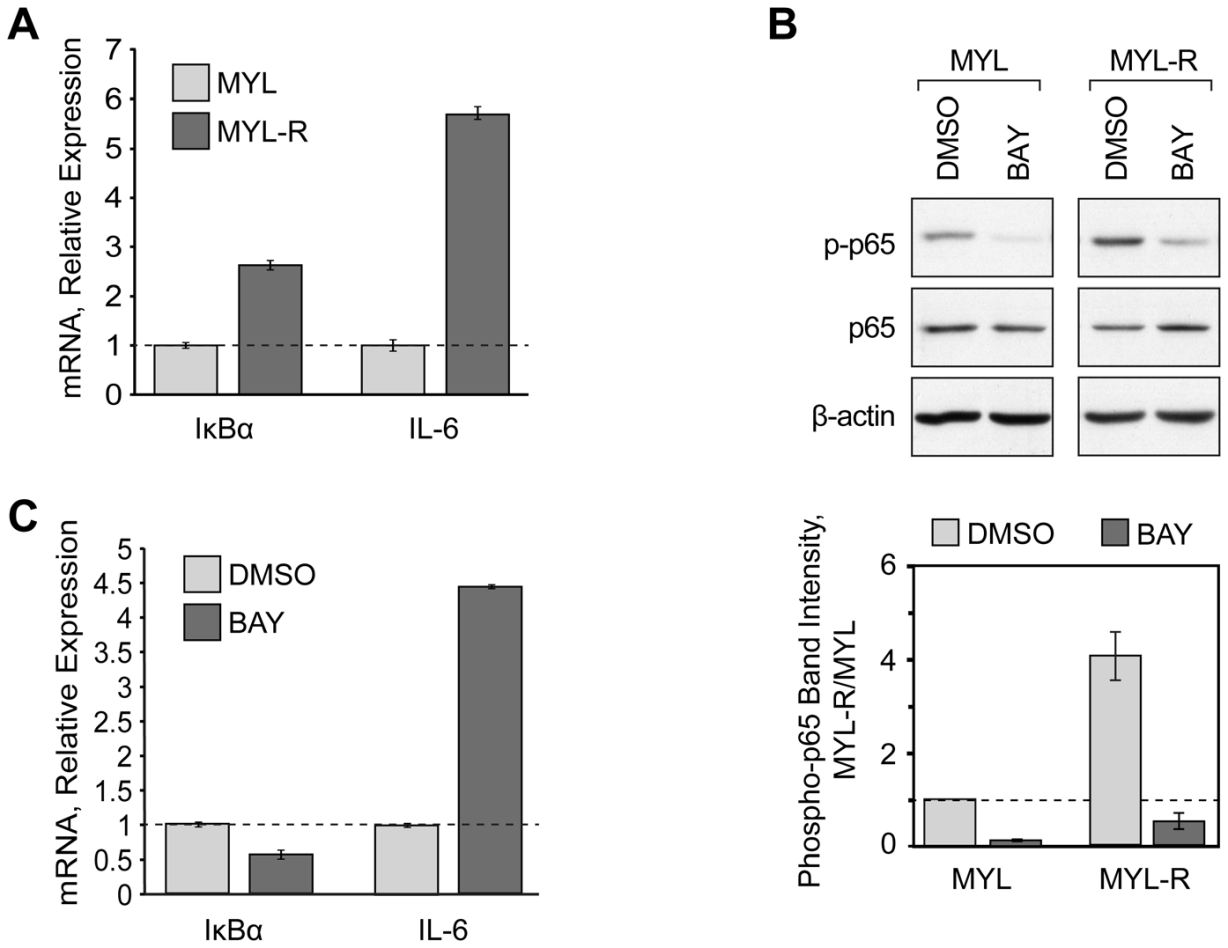
MYL-R cells exhibit increased NF-κB signaling. (**A**) Total RNA was isolated from MYL and MYL-R cells and the expression of NF-κB target genes was evaluated by qRT-PCR. *Error bars*, SE (N = 3). (**B**) MYL and MYL-R cells were treated for two hours with DMSO or the IKK inhibitor BAY 65-1942 (BAY, 10 µM) and then analyzed by immunoblot using the antibodies indicated. The entire blot is shown in Figure S5. *Error bars*, SE (N = 3). (**C**) MYL-R cells were treated for 12 hours with DMSO or BAY (10 µM) and total RNA was isolated and evaluated by qRT-PCR. *Error bars*, SE (N = 3).

IKKα is a subunit of the IKK complex, which serves to activate the transcription factor NF-κB, but can also phosphorylate other proteins involved in signaling and transcriptional regulation [51]–[57]. To establish whether elevated IKKα activity correlated with increased NF-κB activity in MYL-R cells, we isolated total RNA from MYL and MYL-R cells and evaluated the mRNA levels of two established NF-κB target genes, IκBα and IL-6 [58], using real-time quantitative RT-PCR (qRT-PCR). Both IκBα and IL-6 were overexpressed 2.6- and 5.7-fold, respectively, in MYL-R relative to the MYL cells (Figure 4A), consistent with increased NF-κB signaling in MYL-R cells. To investigate the role of IKKα in NF-κB activation in MYL-R cells, we examined the phosphorylation of the NF-κB p65 subunit at Ser536, an IKK phosphorylation site and marker of NF-κB activity. As shown in Figure 4B, basal phosphorylation of p65 was elevated ≈ 5-fold in MYL-R cells compared to MYL cells. Furthermore, p65 phosphorylation was blocked by treating cells with a selective IKK inhibitor, BAY 65-1942 [59].

To further investigate the ability of BAY 65-1942 to inhibit NF-κB signaling in MYL-R cells, we treated cells with the inhibitor (10 µM) for 12 hours and then measured IκBα and IL-6 mRNA expression by qRT-PCR analysis as before. MYL-R cells treated with BAY 65-1942 showed ≈ 2-fold decrease in IκBα expression compared to the DMSO-treated control cells (Figure 4C). Surprisingly, BAY 65-1942 had the opposite effect on IL-6 expression, leading to a 4.4-fold increase over vehicle-treated cells (Figure 4C).

### Kinome Response to Targeted MEK and IKK Inhibitor Treatment

**Figure 5:**
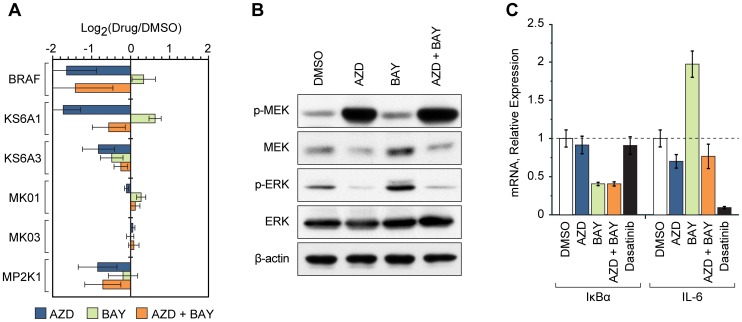
Kinome response to targeted MEK and IKK inhibitor treatment. (**A**) MYL-R cells were treated for 24 hours with AZD6244 (AZD, 5 µM), BAY 65-1942 (BAY, 10 µM) or AZD (5 µM) plus BAY (10 µM), and kinases were isolated and quantified by MIB/MS in two independent experiments. The relative abundances (Drug/DMSO) of kinases in the MEK/ERK pathway are shown. *Error bars*, SE (N = 2). (**B**) MYL-R cells were treated for 24 hours with AZD (5 µM), BAY(10 µM) or AZD (5 µM) plus BAY (10 µM), and were analyzed by immunoblot using the antibodies indicated. Data are representative of three separate experiments. (**C**) MYL-R cells were treated for 12 hours with AZD (5 µM), BAY (10 µM), AZD (5 µM) plus BAY (10 µM), or dasatinib (1 nM). Total RNA was isolated and the expression of IκBα and IL-6 were evaluated by qRT-PCR. *Error bars*, SE (N = 2).

Since our data showed that both the MEK and IKK pathways were activated in MYL-R cells, we used MIB/MS and immunoblot analysis to examine the kinome response to targeted inhibition of these pathways, individually and in combination. Inhibitors of MEK (AZD6244) and IKK (BAY 65-1942) were used at their IC_50_ concentrations, as determined by a 48 hour MTS assay, which achieved sufficient inhibition of kinase activity (as shown in Figures 4B, 5B). As such, MYL-R cells were treated for 24 hours with AZD6244 (5 µM), BAY 65-1942 (10 µM), or a combination of these inhibitors at the same concentrations. To establish the MYL-R kinome response to targeted MEK and IKK inhibition, lysates from these cells were analyzed by MIB/MS as described earlier, and kinase abundance ratios were calculated for each drug treatment compared to DMSO control. In two independent experiments, 111 kinases were quantified (Figure S6, Table S3). Comparing the kinases that were differentially affected by either MEK or IKK inhibition revealed multiple changes in the MIB/MS capture of kinases in the MEK/ERK pathway (Figure 5A). MEK inhibition resulted in significant decreases in the retention of B-Raf (0.32-fold), MEK1 (0.55-fold) and RSK1 (0.30-fold) as expected. Conversely, IKK inhibition resulted in significant increases in B-Raf (1.26-fold) and RSK1 (1.54-fold) bound to MIBs. Treating cells with a combination of the two inhibitors reversed the effects of BAY 65-1942 and decreased B-Raf (0.37-fold), MEK1 (0.61-fold) and RSK1 (0.68-fold) binding. Thus, the MIB/MS analysis of the MYL-R kinome response to targeted IKK inhibition revealed an unexpected increase in the MEK/ERK pathway that was negated by simultaneous inhibition of both kinases.

To confirm the increased MEK/ERK activity in MYL-R cells in response to BAY 65-1942, we treated cells with MEK and IKK inhibitors for 24 hours and analyzed cell lysates by immunoblotting. As shown in Figure 5B, treatment of cells with AZD6244 (5 µM) did not decrease phosphorylation of MEK but induced a significant loss in ERK phosphorylation. In agreement with the MIB/MS results, treatment of cells with BAY 65-1942 (10 µM) increased MEK/ERK activation as shown by a significant increase in ERK phosphorylation. By contrast, the combination of AZD6244 (5 µM) and BAY 65-1942 (10 µM) resulted in an overall decrease in ERK phosphorylation.

Since activation of MEK/ERK has been reported to increase IL-6 production [60], we investigated whether treating cells with a combination of MEK and IKK inhibitors would reverse the increase in IL-6 expression induced by IKK inhibition. MYL-R cells were treated for 24 hours with AZD6244 (5 µM), BAY 65-1942 (10 µM) or dasatinib (1 nM) and RNA was isolated in order to measure IκBα and IL-6 expression by qRT-PCR (Figure 5C). Incubation with BAY 65-1942 resulted in ≈ 2-fold decrease in IκBα mRNA expression, either alone or in combination with AZD6244. IκBα mRNA expression was not affected by AZD6244 treatment nor with dasatinib treatment, in agreement with the modest effects of dasatinib on IKKα phosphorylation (Figure 3). Consistent with our observation that BAY 65-1942 activated MEK, IL-6 mRNA expression was increased ≈ 2-fold after BAY 65-1942 treatment and this increase was prevented by co-incubation with AZD6244. Dasatinib also significantly reduced IL-6 expression, consistent with inhibition of MEK by dasatinib. These results indicate that activation of the MEK/ERK pathway in response to BAY 65-1942, as predicted by MIB/MS, is responsible for increased IL-6 production and can be prevented by the combined inhibition of MEK and IKK.

### Targeted Inhibition of Kinases Detected by MIB/MS Leads to Induction of Apoptosis

**Figure 6:**
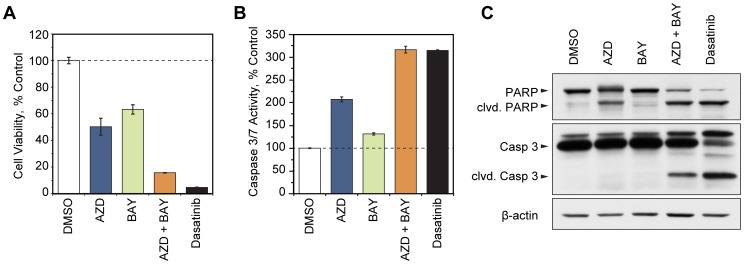
Targeted inhibition of kinases detected by MIB/MS leads to induction of apoptosis. (**A**) MYL-R cells were treated for 48 hours with AZD6244 (AZD, 5 µM), BAY 65-1942 (BAY, 10 µM), AZD (5 µM) plus BAY (10 µM), or dasatinib (1 nM) and cell viability was assessed by MTS assay. *Error bars*, SE (N = 3). (**B**) MYL-R cells were treated for 24 hours with AZD (5 µM), BAY (10 µM), AZD (5 µM) plus BAY (10 µM), or dasatinib (1 nM) and caspase 3/7 activity was assessed by fluorometric assay. *Error bars*, SE (N = 2). (**C**) MYL-R cells were treated for 48 hours with AZD (5 µM), BAY (10 µM), AZD (5 µM) plus BAY (10 µM), or dasatinib (1 nM) and cell lysates were analyzed by immunoblot using the antibodies indicated. Data are representative of two separate experiments.

Our data suggested that the combined inhibition of both MEK and IKK prevented much of the response observed after individual MEK or IKK inhibition. We next asked whether combined inhibition of MEK and IKK would be more effective at inducing cell death than inhibition of either kinase alone. The effects of combined kinase inhibition on MYL-R cell viability was determined by MTS assay. Cells in exponential growth and were treated with AZD6244 (5 µM), BAY 65-1942 (10 µM), a combination of both, or 1 nM dasatinib. Drug and media were replenished after 24 hours, and cell viability was assayed 48 hours after initial treatment according to standard MTS assay procedure. As shown in Figure 6A, treatment with AZD6244 and BAY 65-1942 reduced MYL-R cell viability by ≈ 50% and 37%, respectively, compared to DMSO-treated cells, whereas combined treatment with AZD6244 plus BAY 65-1942 reduced cell viability by ≈ 84%. As a comparison, dasatinib treatment led to a nearly 95% reduction in MYL-R cell viability, consistent with inhibition of Lyn in these cells.

Drug synergy was assessed using CompuSyn software to calculate a combination index (CI) of drug effects at various doses, according to the Chou-Talalay median-effect principle (where CI <1 indicates synergism) [61]–[63]. MYL-R cells were treated with three-fold serial dilutions of AZD6244 and BAY 65-1942, singly or in combination at a constant ratio, and MTS assays were performed as above. AZD6244 and BAY 65-1942 demonstrated synergistic inhibition of cell viability at the dose combination used in Figure 2A (5 µM AZD6244+10 µM BAY 65-1942), which correlates with IC_75_ (CI = 0.48±0.01). Synergism was also indicated at the IC_50_ (CI = 0.56±0.09) and IC_90_ (CI = 0.46±0.02) dose combinations reported by the software. (CI values are the mean of three independent experiments, ± standard deviation.)To determine whether targeted inhibition of MEK and IKK increased apoptosis we assayed inhibitor treated cells for caspase-3/7 activation. MYL-R cells were treated as described above and after 24 hours cells were harvested and lysed with CHAPS buffer in order to gently release activated caspases. Caspase 3/7 activity was measured by incubating cell lysates with a caspase 3/7 substrate (Ac-DEVD-AMC) and measuring the amount of AMC liberated by fluorimetry. As shown in Figure 6B, AZD6244 and BAY 65-1942 treatment induced 2- and 1.3-fold caspase 3/7 activation, respectively, compared to the DMSO-treated cells. Treatment with a combination of AZD6244 plus BAY 65-1942 led to a 3.2-fold increase in caspase 3/7 activity, which was comparable to the amount of caspase 3/7 activation induced by dasatinib.

To confirm that the targeted kinase inhibition resulted in MYL-R apoptosis, we analyzed the levels of cleaved poly(ADP-ribose) polymerase (PARP) by immunoblotting (Figure 6C). MYL-R cells were treated as before and harvested after 48 hours, the same time point at which cell viability was measured. This analysis showed the presence of PARP cleavage in cells treated with AZD6244 alone, however cells treated with a combination of AZD6244 and BAY 65-1942 or with dasatinib showed increased PARP cleavage and a significant loss of total PARP, indicating that the apoptotic process had progressed further with these two treatments. Additionally we tested for the presence of cleaved caspase 3. The results in Figure 6C confirm that treating MYL-R cells with a combination of AZD6244 and BAY 65-1942 was nearly as effective as dasatinib in activating caspase activity, and was significantly more effective than treating with either inhibitor alone.

These results demonstrate that the MIB/MS kinome profile identified MEK and IKK as important mediators of survival, and that combined inhibition of MEK and IKK effectively induced apoptotic cell death in drug-resistant CML. We have also observed increased MEK and IKK phosphorylation that correlated with Lyn amplification and activation in the HL-60 acute myeloid leukemia cell line differentiated with all-*trans* retinoic acid (ATRA) (Figure S7). ATRA-differentiated HL-60 cells exhibit resistance to cytotoxic drugs and death receptor-mediated cell death, and have increased Lyn expression which is required for their survival [64]–[67]. This raises the possibility that upregulation of the MEK/ERK and IKK pathways may play a role in ATRA-induced drug resistance downstream of Lyn, however further studies are required to show a causal relationship between these events.

## Discussion

Herein we describe the application of a recently developed kinase affinity technology (MIB/MS) to investigate kinome adaptations in an imatinib-resistant CML cell line. Our ultimate aim was to develop and apply this technology to obtain insight into the molecular adaptations of drug-resistant cells with the goal of using this information to rationally target kinases contributing to imatinib resistance. Using multiple, structurally distinct kinase inhibitors, this MALDI-TOF/TOF MS based technology provides a high throughput, quantitative approach to interrogate the kinome as described earlier [35]. Importantly, these studies demonstrated that kinase binding to MIBs was a function of both activity and expression, hence MIBs can be used to profile the “activation state” of the kinome. Our studies confirm this and show the utility of the MIB/MS approach to study kinome adaptations in drug-resistant cells and have identified significant quantitative differences in the kinomes of MYL and MYL-R cells (Figure 1, S1). Multiple peptides with 95% confidence were obtained from these samples, allowing the quantification of multiple kinases simultaneously.

Lyn is a SFK with an established role in promoting the survival of imatinib-resistant CML cells from patients and cell lines such as MYL-R independently of BCR-ABL mutations [16], [21]–[23], [25], [68]–[70]. MIB/MS confirmed the increased expression and activation of Lyn in MYL-R cells as reported initially by Ito [22] and others [71]. Using MIB/MS we also detected a substantial number of kinases not previously reported to be increased or decreased in imatinib-resistant cells. In three independent experiments our MIB/MS approach identified and quantified a total of 153 kinases, nearly 50% of the estimated expressed kinome [35], [72]. For the purpose of establishing a MYL-R kinome profile, the significance of these quantifications was established through statistical analysis and only kinase abundance ratios with Benjamini-Hochberg q-values <0.2 were considered to be significantly different.

The MYL-R kinome profile revealed upregulation of multiple kinases involved in cell growth, anti-apoptosis and stress signaling. This included kinases such as MEK2 and ERK2 (MEK/ERK pathway), IKKα (NF-κB pathway) and others NEK9 (mitotic regulation), PRPK (TP53-activating kinase), AAKG1 (AMP-activated protein kinase), RIPK2 (innate immune response) and PRKDC (DNA-dependent protein kinase/DNA damage response). The increased binding of MEK2 and IKKα to MIBs was confirmed to be activity dependent by two independent criteria. First, a greater amount of the phosphorylated kinases was captured on MIBs as determined by immunoblotting and second, this binding was reversed by phosphatase treatment of the samples (Figure S2). These studies illustrate that kinase capture measured by MIB/MS is both a function of changes in kinase expression and kinase activation as reported earlier [35]. In support of a pivotal role for Lyn in MYL-R cells, treatment with dasatinib, a Lyn and SFK inhibitor, prevented the binding of a large number of these kinases to MIBs (Figure S4). Further evidence for Lyn as a regulator of the MEK/ERK pathway was supported by our shRNA data and is consistent with earlier observations demonstrating Lyn as an activator of MEK [45]. By contrast, the mechanism by which Lyn regulates IKKα or other kinases in MYL-R cells (NEK9, PRPK etc.) remains to be elucidated. We also detected a significant increase in PKCβ activity, both by MIB/MS and by immunoblotting. PKCβ has been shown to regulate anti-apoptotic responses in myeloid leukemias [73], however inhibition of PKCβ with bryostatin did not affect the viability of MYL-R cells (unpublished observations). Interestingly, a recent proteomics study profiling kinase expression in drug-refractory head and neck squamous cell carcinoma identified a number of the same kinases (Lyn, MEK, NEK9) as we did in MYL-R cells, suggesting that these may represent a drug resistance kinome profile [74].

Considerable insight may also be obtained from the MIB/MS analysis of the kinases decreased in MYL-R cells. Approximately twice as many kinases were decreased as increased in the MYL-R cells and this was confirmed by both iTRAQ and SILAC quantification methods. Reduced levels of some of these kinases may be expected given that they are direct targets for inhibition by imatinib (Abl, c-Kit) and MYL-R cells were generated by continuous exposure of MYL cells to imatinib [22]. Interestingly, the decreased binding of JNK (MK08) and kinase regulators of JNK [ASK1 (M3K5), HGK (M4K4), ZAK (MLTK)], indicate a decrease in this pro-apoptotic regulatory pathway in MYL-R cells [75], [76]. Down-regulation of these kinases could potentially contribute to the anti-apoptotic properties of MYL-R cells. Decreased NDKM (nucleoside diphosphate kinase) or dCK (deoxycytidine kinase) may also contribute to the reduced sensitivity of MYL-R cells to nucleoside analogs (gemicitabine, Ara-C) that we observed previously (unpublished observations). The marked reduction of ATM may result from the reduced BCR-Abl protein in MYL-R cells as ATM has been shown to directly interact with Abl kinase [77], [78], however the effect of this on cell survival is unclear.

NF-κB plays a key role in regulating anti-apoptotic reactions and responses to chemotherapy [79]. Because we detected increased IKKα and NF-κB signaling in MYL-R cells we examined the specific effects of targeting this pathway. BAY 65-1942 is a selective inhibitor of IKK (alpha and beta isoforms) and an inhibitor of NF-κB responses [59], [80], [81]. While BAY 65-1942 effectively blocked IκBα expression as expected, it stimulated a surprising increase in IL-6 expression in MYL-R cells that correlated with increased ERK phosphorylation. MIBs analysis of MYL-R cells treated with BAY 65-1942 confirmed the activation of the MEK/ERK pathway and an increase in B-Raf, ERK (MK01) and RSK (KS6A1) binding was detected (Figure S6). Since our results suggested that BAY 65-1942 triggered a compensatory activation of the MEK/ERK pathway, we examined the effects of co-targeting these pathways. The MIB/MS analysis of the response to BAY 65-1942 and the MEK inhibitor AZD6244 was complex, with many kinases significantly lowered, including B-Raf, MEK (MAP2K1), and RSK1 (KS6A1) after combination treatment (Figures 5A, S6). Importantly, the combination of these inhibitors not only prevented the BAY 65-1942-stimulated increase in IL-6 and phospho-ERK, but substantially reduced cell viability and increased apoptosis as determined by PARP cleavage and caspase 3/7 activation. Thus these studies demonstrate that MIB/MS profiling provided an experimental rationale for co-targeting the IKK and MEK/ERK pathways and provided insight into why combined inhibition was synergistic compared to inhibition of MEK or IKK alone.

The IKK and MEK/ERK pathways are both frequently dysregulated in cancer [79], [82], and combined targeted inhibition has been shown to synergistically inhibit cell viability [83]. We have demonstrated here that upregulation of these pathways contributes to acquired drug resistance of MYL-R cells through Lyn-dependent signaling (Figures 3, 6), however the commonality of this mechanism of drug resistance in other models remains to be determined. Although upregulation of Lyn in ATRA-differentiated HL-60 cells correlated with increased IKK and MEK phosphorylation (Figure S7), a causal role of these pathways in the drug resistance of these cells has not yet been established.

In summary, the results presented here demonstrate a unique approach to comprehensively determine kinome changes in cells with acquired drug resistance. These methods are expected to be widely applicable to other cell models and we have recently obtained kinome profiles from myeloid cells purified directly from CML or AML patient blood (unpublished observations). While previous studies have identified the importance of Lyn in imatinib-resistant CML [21], [22], [69], our studies further identified dysregulation of two well characterized kinase pathways (MEK/ERK and IKK/NF-κB) and demonstrated their relationship to activation of Lyn. Moreover, we identified significant changes in a number of less well-studied kinases including NEK9, RIPK2, PRPK and PRKDC. Further studies will be necessary to elucidate the importance of these kinases in the development of acquired drug resistance.

## Materials and Methods

### Cell Culture and Reagents

MYL and MYL-R human CML cells were a generous gift from Dr. Hideo Tanaka (Department of Haematology and Oncology, Hiroshima University, Hiroshima, Japan) [22]. HL-60 and HEK 293T cells were obtained from the UNC Lineberger Tissue Culture Facility (Chapel Hill, NC). Cells were cultured in RPMI 1640 medium (Thermo Scientific, Rockford, IL) supplemented with 10% fetal bovine serum (Atlanta Biologicals, Norcross, GA) and 1% antibiotic/antimycotic (Invitrogen, Carlsbad, CA) and were maintained at 37°C in a 5% CO_2_ humidified atmosphere. For SILAC labeling, MYL and MYL-R cells were grown for at least six doublings in SILAC medium, as previously described [35]. For HL-60 cell differentiation, cells were seeded at 1 x 10^5^ cells/mL and then were returned to the incubator for 24 hours. The following day, cells were treated with 200 nM all-*trans* retinoic acid (ATRA) or DMSO, and then were returned to the incubator for an additional 72 hours before harvesting and immunoblot analysis. Reagents were obtained from the following sources: imatinib and dasatinib were from LC Laboratories (Woburn, MA); BAY 65-1942 was from Theralogics, Inc. (Chapel Hill, NC); AZD6244 was synthesized as described previously [35]; TNFα was from Promega (Madison, WI); ATRA was from Sigma-Aldrich (St. Louis, MO).

### Multiplexed Inhibitor Bead Affinity Chromatography

Isolation of kinases from MYL and MYL-R cell lysates was performed as described previously [31], [35]. Briefly, cells were lysed on ice in MIB lysis buffer [50 mM HEPES (pH 7.5), 0.5% Triton X-100, 150 mM NaCl, 1 mM EDTA, 1 mM EGTA, 10 mM sodium fluoride, 2.5 mM sodium orthovanadate, 1X protease inhibitor cocktail (Roche), and 1% of phosphatase inhibitor cocktail 3 (Sigma-Aldrich)]. Cell lysates were sonicated 3×10 seconds on ice and centrifuged at 10,000×g for 10 minutes at 4°C. The supernatant was collected and syringe-filtered through a 0.2 µm SFCA membrane. The filtered lysate (approximately 10-15 mg of protein per experiment) was brought to 1 M NaCl and passed through a column of layered inhibitor-conjugated beads (MIBs) consisting of Sepharose-conjugated Bisindoylmaleimide-X, Dasatinib, Purvalanol B, PP58 and VI16832 [30]. The MIBs were washed with 20 mL of high-salt buffer and 20 mL of low-salt buffer [50 mM HEPES (pH 7.5), 0.5% Triton X-100, 1 mM EDTA, 1 mM EGTA, and 10 mM sodium fluoride, and 1 M NaCl or 150 mM NaCl, respectively]. The columns were washed a final time with 1 mL 0.1% SDS before elution in 1 mL of 0.5% SDS (100°C, 5 min). Eluted kinases were reduced (dithiothreitol) and alkylated (iodoacetamide) prior to being concentrated with Amicon Ultra centrifugal filters (Millipore) and detergent was removed from the concentrated eluate by chloroform/methanol extraction. Protein pellets were resuspended in 50 mM HEPES (pH 8.0) and were digested for 24 hours with sequencing grade modified trypsin (Promega). Digested peptides were labeled with iTRAQ reagent (AB SCIEX, Framingham, MA) for 2 hours in the dark, according to the manufacturer’s instructions. Labeled peptides were cleaned using PepClean C18 spin columns (Thermo Scientific) before fractionation on a Tempo™ LC MALDI Spotting System (AB SCIEX) using a Chromolith® CapRod® RP-18e HR analytical column (Merck KGaA, Darmstadt, Germany).

### MS Analysis

MS and MS/MS data were acquired on a MALDI TOF/TOF 5800 or 4800 (AB SCIEX). MS/MS spectra were searched against the Uniprot/Swiss-Prot database with ProteinPilot™ software version 3.0 (AB SCIEX) (Paragon algorithm 3.0.0.0, 113442) for peptide and protein identifications. Protein identifications were accepted with an Unused ProtScore >1.3 (corresponding to >95% confidence). Relative quantification of kinases bound to MIBs was determined with ProteinPilot™ using auto bias-correction and background correction.

### Statistical Analysis of MIB/MS Kinase Quantification

We performed three independent MIB/MS experiments to profile the kinomes of MYL and MYL-R cells and quantified a total of 153 unique kinases. For each kinase, we computed the pooled protein ratio and p-value across the three replicates as follows. Let *y_ij_* denote the Log2 protein ratio for kinase *i*, *i* = 1,…,153 in replicate *j*, *j* = 1,2,3; and let *w_ij_* denote the number of peptides used for calculation of the protein ratio for kinase *i* in replicate *j*. The pooled protein ratio for kinase *i* is defined as 2*^yi^*, where 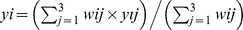. To avoid directional conflict, we convert the two-sided p-values reported in ProteinPilot™ to one-sided p-values and denote it as *p_ij_*. We applied Stouffer’s Z-score method to combine the p-values. Let *z_ij_* = Φ^−1^(1-*p_ij_*), where Φ is the standard Gaussian cumulative distribution function. Define the combined Z-score as 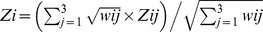. The combined two-sided p-value for kinase *i* is given as *p_i_ = *2(1-Φ(|*Z_i_*|)). For each replicate, we identified kinases that exhibit statistically significant changes in expression based on Benjamini-Hochberg adjusted p-values at FDR of 0.2 to account for multiple comparisons. To profile the MYL-R kinome response to kinase inhibitor treatment we performed two independent MIB/MS experiments and three technical replicates of the second experiment. Kinase abundance ratios and p-values were pooled as described above.

### Immunoblot Analysis

Cells were collected and lysed using modified RIPA buffer (150 mM NaCl, 9.1 mM Na_2_HPO_4_, 1.7 mM NaH_2_PO_4_, 1% NP-40, and 0.5% deoxycholic acid; adjusted to pH 7.4) supplemented with protease inhibitor cocktail (Roche Diagnostics, Mannheim, Germany), 1 mM sodium orthovanadate_,_ and phosphatase inhibitor cocktail 3 (Sigma-Aldrich). The lysate was centrifuged and the protein concentration was determined using Bradford reagent (Thermo Scientific). Gel samples were prepared by adding 20–40 µg protein lysate to Laemmli sample buffer [final concentration: 0.25 M Tris (pH 6.8), 10% glycerol, 5% β-mercaptoethanol, 0.001 µg/mL Bromphenol blue] and the samples were separated by SDS-polyacrylamide gel electrophoresis and then transferred to a PVDF membrane (Millipore, Billerica, MA). Membranes were blocked with 5% non-fat dry milk or 5% bovine serum albumin in Tris-buffered saline supplemented with Tween-20 (TBS-T) [10 mM Tris-HCl (pH 7.6), 150 mM NaCl, 0.05% Tween-20] for 1 hour at room temperature. Membranes were incubated with primary antibodies for 1 hour at room temperature or overnight at 4°C, followed by incubation with secondary antibodies for 1 hour at room temperature, and were then developed using Amersham ECL Western blotting detection reagents (GE Healthcare, Piscataway, NJ). Densitometry was performed using ImageJ (NIH). Antibodies were from the following sources: BCR-Abl, p-SFK (Y416), p-IKKα/β, p-MEK1/2, MEK1/2, p-ERK1/2, ERK1/2, JNK1, JAK1, RIPK2, p-p65 (S536), p65, PARP, cleaved PARP, caspase 3 and cleaved caspase 3 were from Cell Signaling Technology (Danvers, MA); IKKα, IKKβ and ASK1 were from Millipore; β-actin, ATM, c-KIT, Lyn, p-PKCβ (T642), PKCβ, ERK2, FAK, MLTK, Yes, GSK3α and CDK2 were from Santa Cruz Biotechnology (Santa Cruz, CA); dCK and NEK9 were from Abcam (Cambridge, MA); FAK (Y397) was from BioSource International (Life Technologies, Grand Island, NY); Anti-mouse and anti-rabbit IgG-HRP conjugated secondary antibodies were from Promega. Primary antibodies were diluted at 1∶1,000 in 5% bovine serum albumin in TBS-T with the exception of Lyn (1∶10,000). Secondary antibodies were diluted at 1∶10,000 or 1∶15,000 in 5% dry, non-fat milk in TBS-T.

### MIBs Pull-down Assay

MYL and MYL-R cells were harvested and lysed for 20 minutes on ice in buffer containing 50 mM Tris-HCl (pH 8.0), 0.5% NP-40, 150 mM NaCl, and 2X protease inhibitor cocktail (Roche). Lysate was cleared by centrifugation at 10,000 x g for 10 minutes at 4°C and protein concentration was determined with Bradford reagent (Thermo Scientific). Kinases were captured by mixing 1 mg protein lysate with 50 µL MIBs at 4°C for 1 hour. MIBs were washed 3 times with lysis buffer, then kinases were eluted with 100 µL 1X Laemmli sample buffer (100°C, 5 min) and analyzed by immunoblotting. To assess the phosphorylation-dependent binding of kinases to MIBs, 0.5 mg of each protein lysate was adjusted to 1 mg/mL with lysis buffer and either treated with 100 units of calf intestinal alkaline phosphatase (New England Biolabs, Ipswich, MA) or, for un-treated samples, supplemented with phosphatase inhibitors. Samples were incubated at 30°C for 30 minutes with mixing, then placed on ice. Kinases were then isolated and analyzed by immunoblot as above.

### shRNA Knock-down of Lyn

pLKO.1 lentiviral vectors containing shRNA directed against Lyn or a non-targeting shRNA were obtained from the UNC Vector Core. Lentiviral packaging vector psPAX2 (Addgene plasmid 12260) and envelope vector pMD2.G (Addgene plasmid 12259) were obtained from Addgene, courtesy of Didier Trono (http://tronolab.epfl.ch/). Lentivirus production and MYL-R cell infection was done according to the protocol supplied by The RNAi Consortium (http://www.broadinstitute.org/rnai/public/resources/protocols). Briefly, HEK 293T cells growing in 15 cm dishes were transfected with 10 µg shRNA transfer vector, 7.5 µg psPAX2 and 2.5 µg pMD2.G. Viral supernatant was collected at 48 and 72 hours post-transfection. MYL-R cells were seeded at 2 x 10^5^ cells/mL in 10 mL growth medium containing 8 µg/mL polybrene and incubated with 1 mL viral supernatant overnight. After 48 hours, transduced MYL-R cells were selected in media supplemented with puromycin (2 µg/mL) for 96 hours and cells were harvested for analysis by immunoblotting.

### qRT-PCR

RNA was isolated from cell lines using TRIzol™ reagent (Invitrogen) according to the manufacturer’s protocol. Reverse transcription was carried out on 5 µg RNA in a 20 µL reaction using SuperScriptII reverse transcriptase (Invitrogen) and random primers, according to the manufacturer’s protocol. The cDNA was analyzed by real-time qPCR using inventoried TaqMan™ Gene Expression Assays (AB Sciex) on an Applied Biosystems 7500 Fast Real-Time PCR System.

### Caspase 3/7 Activity Assay

MYL-R cells (1×10^6^) were incubated with kinase inhibitors for 24 hours, lysed on ice for 20 minutes with 100 µL lysis buffer [50 mM HEPES (pH 7.4), 5 mM CHAPS and 5 mM DTT], then centrifuged at 10,000×g for 10 minutes at 4°C. In a 96-well plate, 10 µL of supernatant was added to 200 µL of assay buffer [20 mM HEPES (pH 7.4), 0.1% CHAPS, 2 mM EDTA, 5 mM DTT and 15 µM Ac-DEVD-AMC caspase 3/7 substrate (Sigma-Aldrich)] and the plate was incubated in the dark for 2 hours at room temperature. The fluorescence of liberated AMC (7-amido-4-methylcoumarin) was measured using a FLUOstar Galaxy plate reader (BMG Labtech, Cary, NC) with a 360 nm excitation filter and a 460 nm emission filter.

### Cell Viability Assay

Cell viability was determined by seeding MYL-R cells on a 96-well plate at 4 x 10^4^ cells/well in 100 µL RPMI growth medium supplemented with kinase inhibitors. Growth media and kinase inhibitors were replenished at 24 hours, and at 48 hours. 20 µL of MTS assay reagent (CellTiter 96 AQueous One Solution Cell Proliferation Assay™, Promega) was added to each well. The plate was returned to the incubator for approximately 1 hour and the absorbance at 490 nm was recorded. For combination index (CI) experiments, cells were grown and assayed as above. To determine AZD6244 and BAY 65-1942 dose-effects, cells were treated with a series of three-fold dilutions of each drug singly, or in combination while maintaining a constant ratio of 1∶2, respectively. Cell viability results were analyzed by CompuSyn software (ComboSyn, Inc., Paramus, NJ) to derive CI values. The CI values from three independent experiments were averaged.

## Supporting Information

Figure S1 **MIB/MS analysis of kinases from MYL and MYL-R cells.** Kinases from MYL and MYL-R cells were analyzed by MIB/MS in three independent experiments. **(A)** Kinase abundance ratios (MYL-R/MYL) pooled from three experimental replicates using iTRAQ for relative quantification. *Dashed line*, ±1.5-fold change; *error bars*, SE (N = 3). **(B)** Comparison of kinase abundance ratios (MYL-R/MYL) obtained using iTRAQ and SILAC for relative quantification. Kinases identified using both methods are shown. *Dashed line*, ±1.5-fold change, *error bars*, SE (N = 2). **(C)** The trend in kinase abundance ratio changes (MYL-R/MYL) using iTRAQ (triangles) compared to SILAC (squares) for relative quantification.
(PDF)

Figure S2 **Kinases bind to MIBs in an activity-dependent manner.** MYL and MYL-R cell lysates were treated with calf instestinal alkaline phosphatase and the amount of IKKα, Lyn and MEK captured by MIBs with and without phosphatase treatment was compared by immunoblot analysis using the antibodies indicated.
(PDF)

Figure S3 **Phospho-IKKα is elevated in MYL-R cells.** Proteins from MYL and MYL-R cell lysates were separated by SDS-PAGE on a 6% polyacrylamide gel and the migrations of IKKα, IKKβ and phospho-IKK were compared by immunoblot analysis using antibodies against IKKα, IKKβ and phospho-IKKα/β.
(PDF)

Figure S4 **MYL-R kinome response to dasatinib treatment.** MYL-R cells were treated for 1 hour with dasatinib (10 nM) or DMSO and changes to the kinome were analyzed by MIB/MS in a single experiment. Data is represented as changes in the kinase abundance ratios induced by dasatinib treatment. A total of 116 kinases were quantified. *Dashed lines*, ±1.5-fold change; *error bars*, SE.
(PDF)

Figure S5 **IKK activity is blocked by the kinase inhibitor BAY 65-1942.** MYL and MYL-R cells were pre-treated for 2 hours with BAY 65-1942 (BAY, 10 µM) or DMSO and then stimulated with TNFα (10 ng/µL) for 5 minutes. The ability of BAY to block phosphorylation of p65 (S536) was analyzed by immunoblot using the antibodies indicated. This is the uncropped blot of that shown in Figure 4B.
(PDF)

Figure S6 **MIB/MS analysis of MYL-R kinome response to targeted inhibition of MEK and IKK.** MYL-R cells were treated for 24 hours with DMSO, AZD6244 (AZD, 5 µM), BAY 65-1942 (BAY, 10 µM), or AZD (5 µM) plus BAY (10 µM) and kinases were analyzed by MIB/MS in two independent experiments. The relative abundances (Drug/DMSO) of 111 quantified kinases are shown. *Dashed lines*, ±1.5-fold change; *error bars*, SE (N = 2).
(PDF)

Figure S7 **HL-60 AML cells differentiated with ATRA show increased phosphorylation of Lyn, IKKα and MEK.** HL-60 cells were treated for 96 hours with all-*trans* retinoic acid (ATRA) (200 nM) or DMSO and cell lysates were analyzed by immunoblot with the antibodies indicated. Data are representative of three separate experiments.
(PDF)

Table S1 **MIB/MS analysis of kinases from MYL and MYL-R cells.** Kinases from MYL and MYL-R cells were analyzed by MIB/MS. Kinase abundance ratios (MYL-R/MYL), p-values and adjusted p-values (q-values) from three independent experiments are shown.
(XLS)

Table S2 **MYL-R kinome response to dasatinib treatment.** MYL-R cells were treated for 1 hour with dasatinib (10 nM) or DMSO, and changes to the kinome were analyzed by MIB/MS. Kinase abundance ratios (Dasatinib/DMSO), p-values and adjusted p-values (q-values) are shown.
(XLS)

Table S3 **MIB/MS analysis of MYL-R kinome response to targeted inhibition of MEK and IKK.** MYL-R cells were treated for 24 hours with DMSO, AZD6244 (5 µM), BAY 65-1942 (10 µM) or AZD6244 plus BAY 65-1942 and kinases were analyzed by MIB/MS. Kinase abundance ratios (Drug/DMSO), p-values and adjusted p-values (q-values) are shown.
(XLS)
