# Supplementary material for: Application of Multiplexed Kinase Inhibitor Beads to Study Kinome Adaptations in Drug-Resistant Leukemia
Source: PLoS One. 2013 Jun 24;8(6):e66755. doi: 10.1371/journal.pone.0066755 (PMC3691232; doi:10.1371/journal.pone.0066755)

Supplementary Figure S1A

Figure S1. MIB/MS analysis of kinases from MYL and MYL-R cells.
Kinases from MYL and MYL-R cells were analyzed by MIB/MS in three independent experiments. **(A)** Kinase abundance ratios (MYL-R/MYL) pooled from three experimental replicates using iTRAQ for relative quantification. *Dashed lines*, ± 1.5-fold change; *error bars* ± SE (N=3). **(B)** Comparison of kinase abundance ratios (MYL-R/MYL) obtained using iTRAQ and SILAC for relative quantification. Kinases identified using both methods are shown. *Dashed lines*, ± 1.5-fold change; *error bars* ± SE (N=2). **(C)** The trend in kinase abundance ratio changes (MYL-R/MYL) using iTRAQ (triangles) compared to SILAC (squares) for relative quantification.

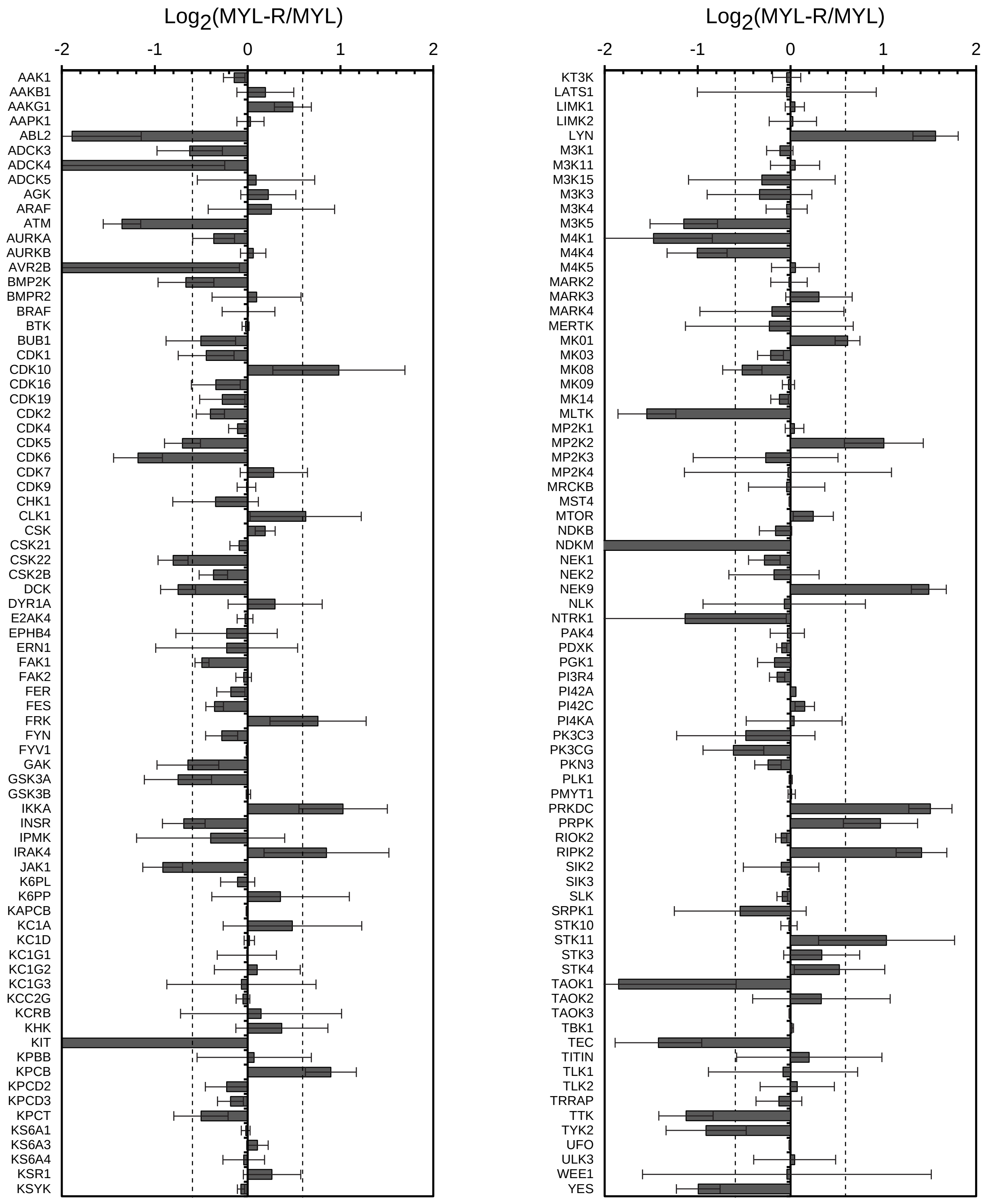

Supplementary Figure S1B

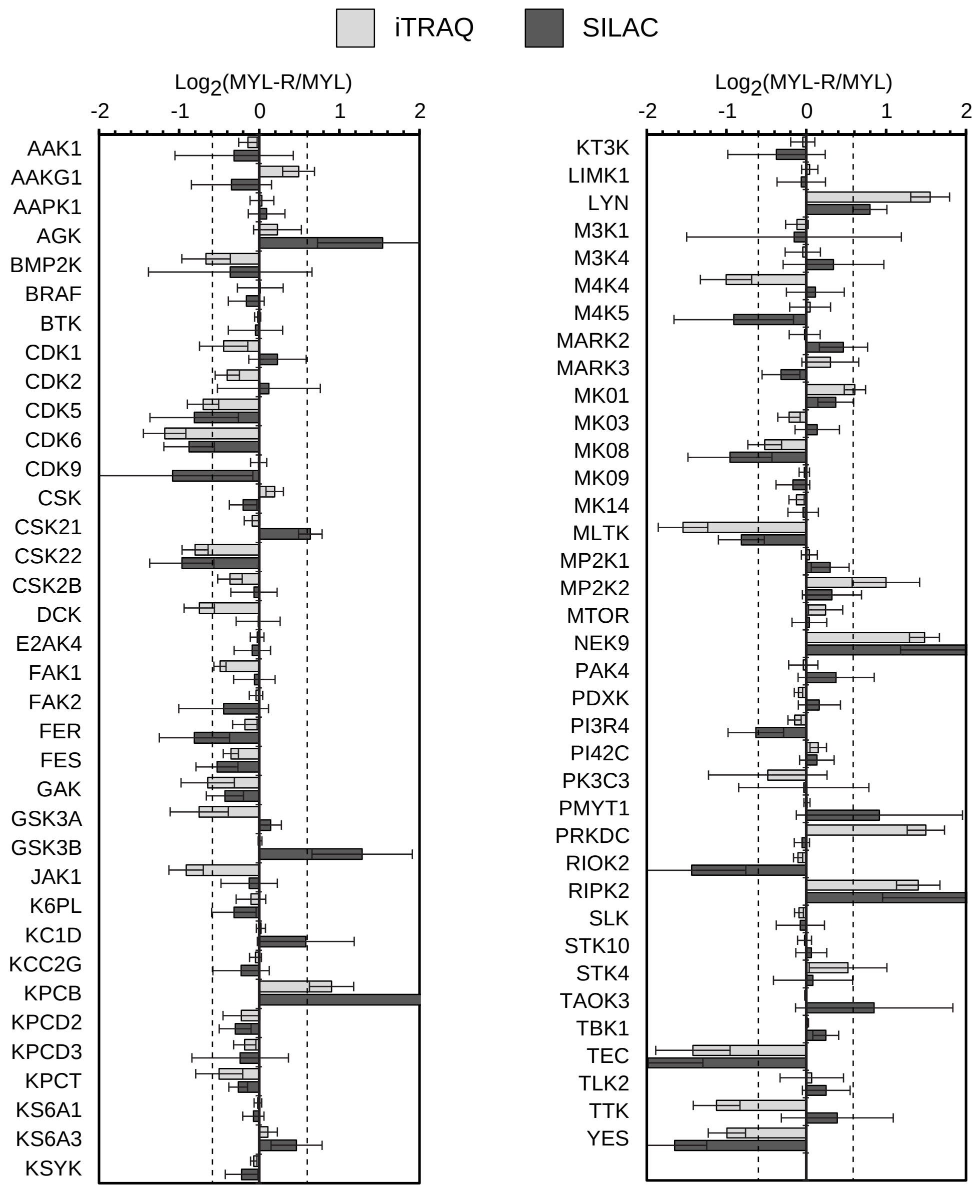

Supplementary Figure S1C

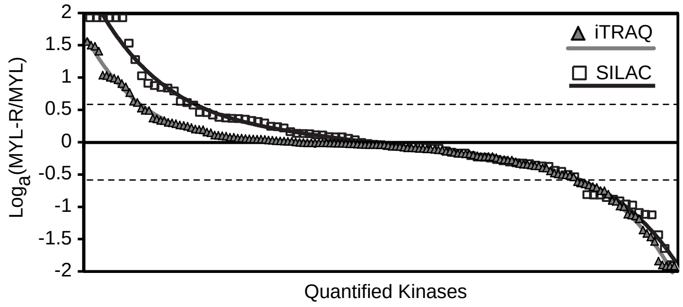

Supplement: Figure S1 — MIB/MS analysis of kinases from MYL and MYL-R cells. Kinases from MYL and MYL-R cells were analyzed by MIB/MS in three independent experiments. (A) Kinase abundance ratios (MYL-R/MYL) pooled from three experimental replicates using iTRAQ for relative quantification. Dashed line, ±1.5-fold change; error bars, SE (N = 3). (B) Comparison of kinase abundance ratios (MYL-R/MYL) obtained using iTRAQ and SILAC for relative quantification. Kinases identified using both methods are shown. Dashed line, ±1.5-fold change, error bars, SE (N = 2). (C) The trend in kinase abundance ratio changes (MYL-R/MYL) using iTRAQ (triangles) compared to SILAC (squares) for relative quantification. (PDF) [file pone.0066755.s001.pdf]
