# Supplementary material for: Application of Multiplexed Kinase Inhibitor Beads to Study Kinome Adaptations in Drug-Resistant Leukemia
Source: PLoS One. 2013 Jun 24;8(6):e66755. doi: 10.1371/journal.pone.0066755 (PMC3691232; doi:10.1371/journal.pone.0066755)

Supplementary Figure S2

Figure S2. Kinases bind to MIBs in an activity-dependent manner.
MYL and MYL-R cell lysates were treated with calf instestinal alkaline phosphatase and the amount of IKKα, Lyn and MEK captured by MIBs with and without phosphatase treatment was compared by immunoblot analysis using the antibodies indicated.

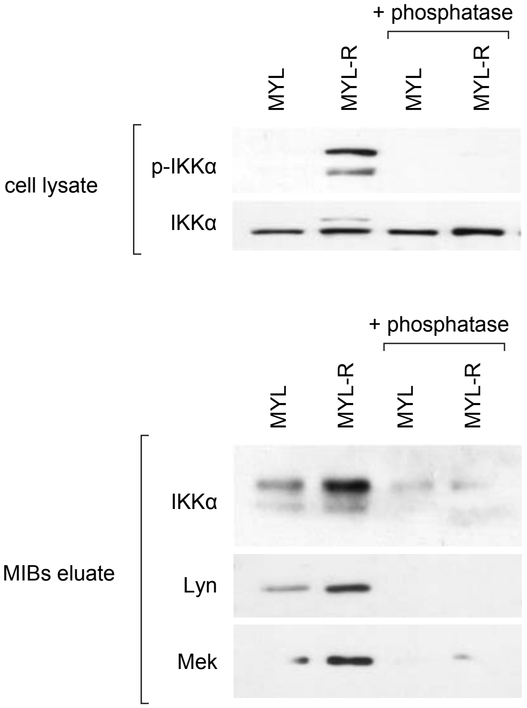

Supplement: Figure S2 — Kinases bind to MIBs in an activity-dependent manner. MYL and MYL-R cell lysates were treated with calf instestinal alkaline phosphatase and the amount of IKKα, Lyn and MEK captured by MIBs with and without phosphatase treatment was compared by immunoblot analysis using the antibodies indicated. (PDF) [file pone.0066755.s002.pdf]
