# Supplementary material for: Application of Multiplexed Kinase Inhibitor Beads to Study Kinome Adaptations in Drug-Resistant Leukemia
Source: PLoS One. 2013 Jun 24;8(6):e66755. doi: 10.1371/journal.pone.0066755 (PMC3691232; doi:10.1371/journal.pone.0066755)

Supplementary Figure S3

Figure S3. Phospho-IKKα is elevated in MYL-R cells.
Proteins from MYL and MYL-R cell lysates were separated by SDS-PAGE on a 6% poly-acrylamide gel and the migrations of IKKα, IKKβ and phospho-IKK were compared by immunoblot analysis using antibodies against IKKα, IKKβ and phospho-IKKα/β.

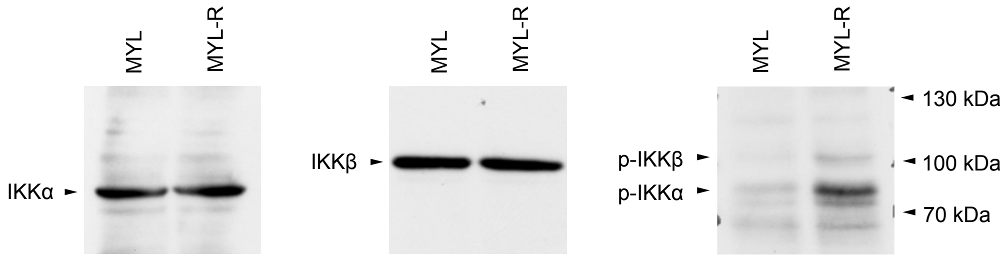

Supplement: Figure S3 — Phospho-IKKα is elevated in MYL-R cells. Proteins from MYL and MYL-R cell lysates were separated by SDS-PAGE on a 6% polyacrylamide gel and the migrations of IKKα, IKKβ and phospho-IKK were compared by immunoblot analysis using antibodies against IKKα, IKKβ and phospho-IKKα/β. (PDF) [file pone.0066755.s003.pdf]
