# Supplementary material for: Application of Multiplexed Kinase Inhibitor Beads to Study Kinome Adaptations in Drug-Resistant Leukemia
Source: PLoS One. 2013 Jun 24;8(6):e66755. doi: 10.1371/journal.pone.0066755 (PMC3691232; doi:10.1371/journal.pone.0066755)

Supplementary Figure S4

Figure S4. MYL-R kinome response to dasatinib treatment.
MYL-R cells were treated for 1 hour with dasatinib (10 nM) or DMSO and changes to the kinome were analyzed by MIB/MS in a single experiment. Data is represented as changes in the kinase abundance ratios induced by dasatinib treatment. A total of 116 kinases were quantified. *Dashed lines*, ± 1.5-fold change; *error bars* ± SE.

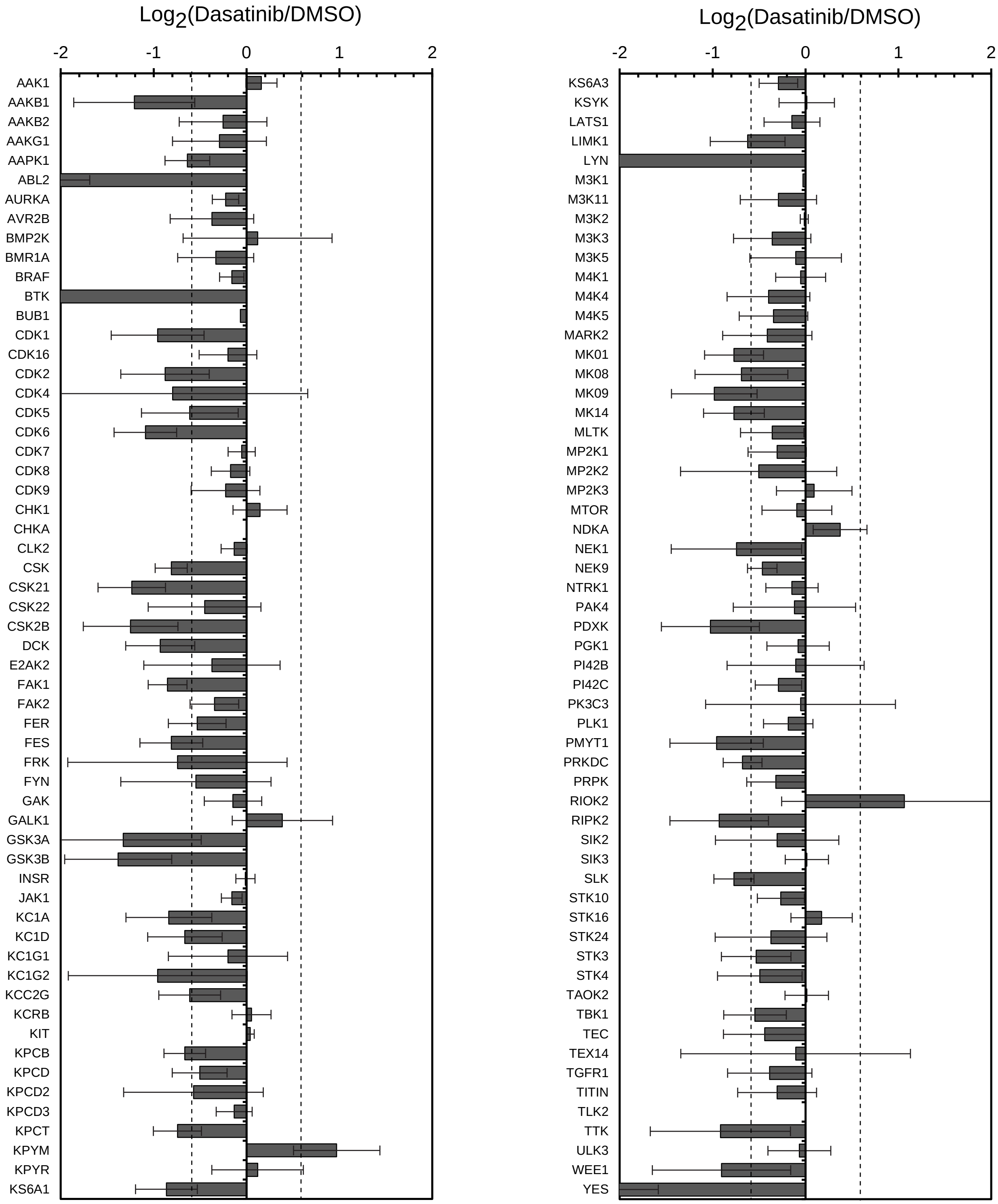

Supplement: Figure S4 — MYL-R kinome response to dasatinib treatment. MYL-R cells were treated for 1 hour with dasatinib (10 nM) or DMSO and changes to the kinome were analyzed by MIB/MS in a single experiment. Data is represented as changes in the kinase abundance ratios induced by dasatinib treatment. A total of 116 kinases were quantified. Dashed lines, ±1.5-fold change; error bars, SE. (PDF) [file pone.0066755.s004.pdf]
