# Supplementary material for: Application of Multiplexed Kinase Inhibitor Beads to Study Kinome Adaptations in Drug-Resistant Leukemia
Source: PLoS One. 2013 Jun 24;8(6):e66755. doi: 10.1371/journal.pone.0066755 (PMC3691232; doi:10.1371/journal.pone.0066755)

Supplementary Figure S5

Figure S5. IKK activity is blocked by the kinase inhibitor BAY 65-1942.
MYL and MYL-R cells were pre-treated for 2 hours with BAY 65-1942 (BAY, 10 μM) or DMSO and then stimulated with TNFα (10 ng/μL) for 5 minutes. The ability of BAY to block phosphorylation of p65 (S536) was analyzed by immunoblot using the antibodies indicated. This is the uncropped blot of that shown in Figure 4B.

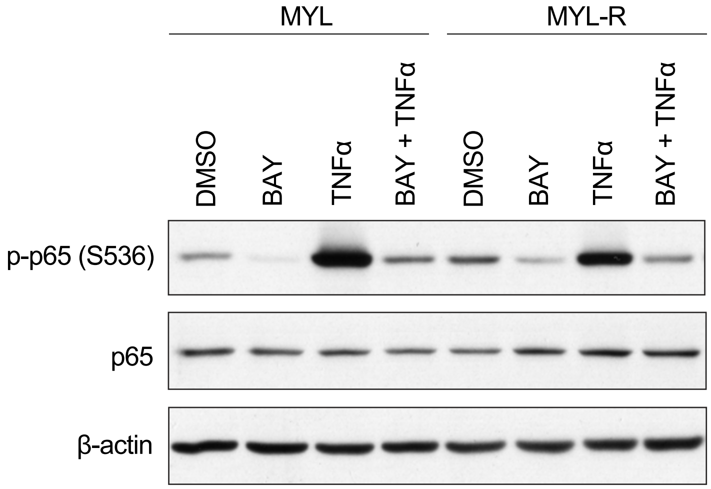

Supplement: Figure S5 — IKK activity is blocked by the kinase inhibitor BAY 65-1942. MYL and MYL-R cells were pre-treated for 2 hours with BAY 65-1942 (BAY, 10 µM) or DMSO and then stimulated with TNFα (10 ng/µL) for 5 minutes. The ability of BAY to block phosphorylation of p65 (S536) was analyzed by immunoblot using the antibodies indicated. This is the uncropped blot of that shown in Figure 4B. (PDF) [file pone.0066755.s005.pdf]
