# Supplementary material for: Application of Multiplexed Kinase Inhibitor Beads to Study Kinome Adaptations in Drug-Resistant Leukemia
Source: PLoS One. 2013 Jun 24;8(6):e66755. doi: 10.1371/journal.pone.0066755 (PMC3691232; doi:10.1371/journal.pone.0066755)

Supplementary Figure S6

Figure S6. MIB/MS analysis of MYL-R kinome response to targeted inhibition of MEK and IKK. MYL-R cells were treated for 24 hours with DMSO, AZD6244 (AZD, 5 μM), BAY 65-1942 (BAY, 10 μM), or AZD (5 μM) plus BAY (10 μM) and kinases were analyzed by MIB/MS in two independent experiments. The relative abundances (Drug/DMSO) of 111 quantified kinases are shown. *Dashed lines*, ± 1.5-fold change; *error bars*, ± SE (N=2).

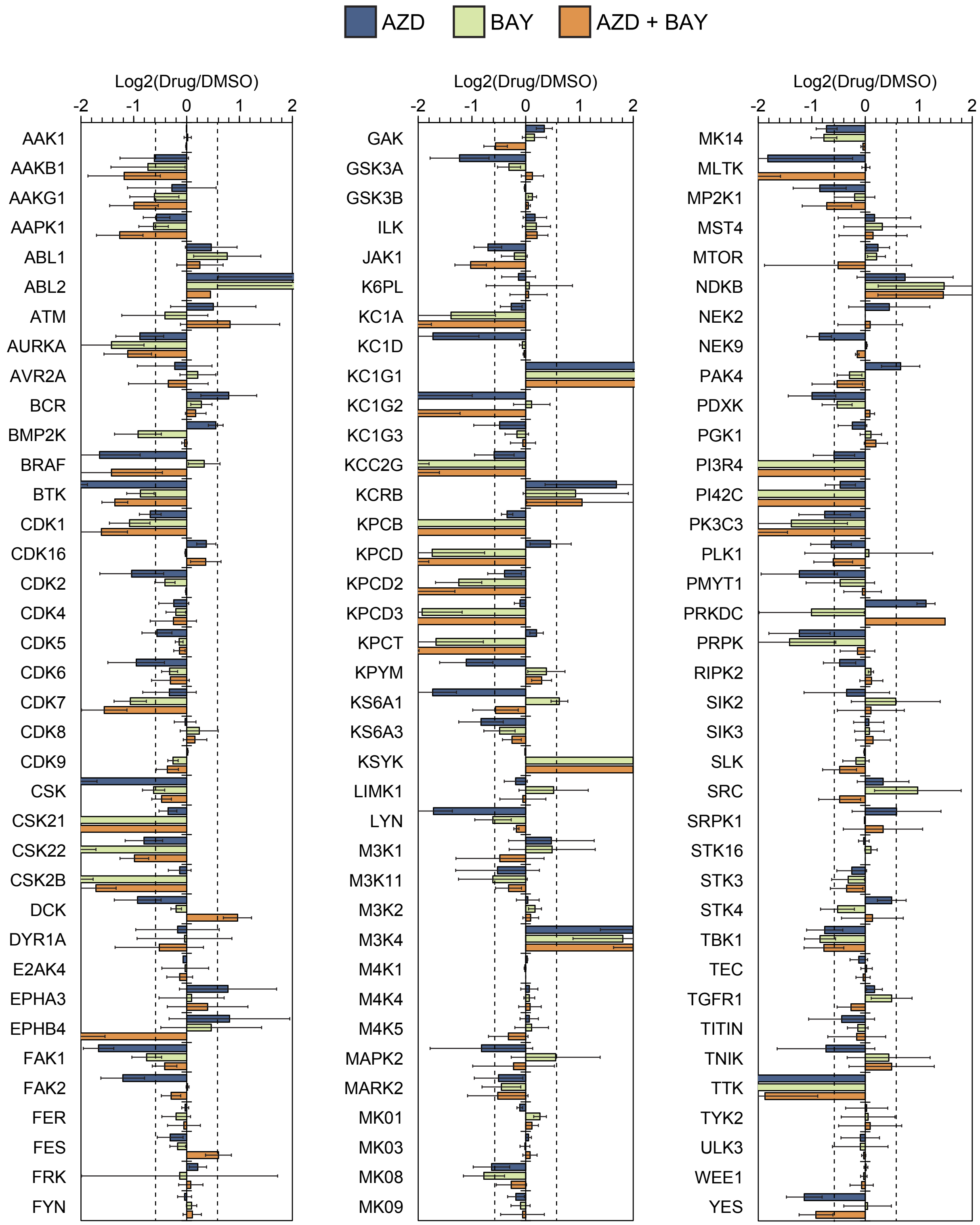

Supplement: Figure S6 — MIB/MS analysis of MYL-R kinome response to targeted inhibition of MEK and IKK. MYL-R cells were treated for 24 hours with DMSO, AZD6244 (AZD, 5 µM), BAY 65-1942 (BAY, 10 µM), or AZD (5 µM) plus BAY (10 µM) and kinases were analyzed by MIB/MS in two independent experiments. The relative abundances (Drug/DMSO) of 111 quantified kinases are shown. Dashed lines, ±1.5-fold change; error bars, SE (N = 2). (PDF) [file pone.0066755.s006.pdf]
