# Supplementary material for: Application of Multiplexed Kinase Inhibitor Beads to Study Kinome Adaptations in Drug-Resistant Leukemia
Source: PLoS One. 2013 Jun 24;8(6):e66755. doi: 10.1371/journal.pone.0066755 (PMC3691232; doi:10.1371/journal.pone.0066755)

Supplementary Figure S7

Figure S7. HL-60 AML cells differentiated with ATRA show increased phosphorylation of Lyn, IKKα and MEK.
HL-60 cells were treated for 96 hours with *all-trans*-retinoic acid (ATRA) (200 nM) or DMSO and cell lysates were analyzed by immunoblot with the antibodies indicated.

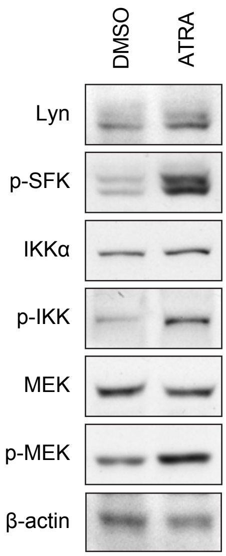

Supplement: Figure S7 — HL-60 AML cells differentiated with ATRA show increased phosphorylation of Lyn, IKKα and MEK. HL-60 cells were treated for 96 hours with all-trans retinoic acid (ATRA) (200 nM) or DMSO and cell lysates were analyzed by immunoblot with the antibodies indicated. Data are representative of three separate experiments. (PDF) [file pone.0066755.s007.pdf]
